# Syntheses and crystal structures of the five- and sixfold coordinated complexes diiso­seleno­cyanato­tris­(2-methyl­pyridine *N*-oxide)cobalt(II) and diiso­seleno­cyanato­tetra­kis­(2-methyl­pyridine *N*-oxide)cobalt(II)

**DOI:** 10.1107/S2056989024005073

**Published:** 2024-06-07

**Authors:** Christian Näther, Inke Jess

**Affiliations:** a Institut für Anorganische Chemie, Universität Kiel, Germany; University of Aberdeen, United Kingdom

**Keywords:** synthesis, crystal structure, trigonal-bipyramidal coordination, cobalt thio­cyanate, 2-methyl­pyridine *N*-oxide

## Abstract

The reaction of CoBr_2_, KNCSe and 2-methyl­pyridine *N*-oxide (C_6_H_7_NO) in ethanol leads to the formation of crystals of [Co(NCSe)_2_(C_6_H_7_NO)_3_] (**1**) and [Co(NCSe)_2_(C_6_H_7_NO)_4_] (**2**) from the same reaction mixture. The asymmetric unit of **1** is built up of one Co^II^ cation, two NCSe^−^ iso­seleno­cyanate anions and three 2-methyl­pyridine *N*-oxide coligands, with all atoms located on general positions. The asymmetric unit of **2** consists of two cobalt cations, four iso­seleno­canate anions and eight 2-methyl­pyridine *N*-oxide coligands in general positions, because two crystallographically independent complexes are present. In compound **1**, the Co^II^ cations are fivefold coordinated to two terminally N-bonded anionic ligands and three 2-methyl­pyridine *N*-oxide coligands within a slightly distorted trigonal–bipyramidal coordination, forming discrete complexes with the O atoms occupying the equatorial sites. In compound **2**, each of the two complexes is coordinated to two terminally N-bonded iso­seleno­cyanate anions and four 2-methyl­pyridine *N*-oxide coligands within a slightly distorted *cis*-CoN_2_O_4_ octa­hedral coordination geometry. In the crystal structures of **1** and **2**, the complexes are linked by weak C—H⋯Se and C—H⋯O contacts. Powder X-ray diffraction reveals that neither of the two compounds were obtained as a pure crystalline phase.

## Abstract 2

The crystal structures of the title compounds, which arose from the same reaction, consist of discrete complexes in which the cobalt cations are either in a CoN_2_O_3_ trigonal–bipyramidal or a CoN_2_O_4_ octa­hedral coordination.

## 1. Chemical context

Numerous crystal structures of transition-metal thio­cyanate coordination compounds have been reported in the literature, which are of inter­est not only because of their versatile structural behavior but also for their magnetic properties. In contrast, much less is known about the corresponding seleno­cyanate coordination compounds. This might originate from the fact that such compounds are frequently less stable and very often more difficult to prepare, especially if the focus is on the synthesis of compounds with bridging anionic ligands, which are of inter­est because of their promising magnetic properties. In this context, compounds based on cobalt are of special inter­est because they can show versatile magnetic behaviors including ferromagnetic ordering or single-chain magnet behavior (Mautner *et al.*, 2018 ▸; Rams *et al.*, 2017 ▸ and 2020 ▸). In this regard we have shown that, for example, exchange of seleno by thio­cyanate anions leads to an increase of the energy barrier and the intra­chain inter­actions (Neumann *et al.*, 2019 ▸).

Concerning the structural behavior of cobalt thio- and seleno­cyanates, in most cases an octhaedral coordination geometry is observed, independent of the question whether discrete complexes or coordination polymers are considered. In rarer cases, especially with strong donor ligands, a tetra­hedral coordination is found, with many examples for thio­cyanates (Mautner *et al.*, 2018 ▸; Neumann *et al.*, 2018 ▸), whereas for seleno­cyanate compounds with additional coligands, no example has been reported. There are only two compounds with the composition CoHg(NCSe)_4_ (Cambridge Structural Database refcode MURQOH; Li *et al.*, 2006 ▸) and [Co(NCSe)_4_]^2−^[(NH_4_)_2_]^+^ (QQQBEY; Kergoat *et al.*, 1970 ▸) that contain no additional coligands and in which the metal cations are either linked into chains or in which discrete complexes are formed. Finally, with Co(NCS)_2_, compounds with a fivefold coordination are rarer than with a fourfold coordination, and with seleno­cyanate only three examples are found. This includes two discrete complexes with tridentate ligands [DUCVEF (Hopa *et al.*, 2020 ▸) and VONXUT (Solanki & Kumar, 2014 ▸)] and one tetra­nuclear complex (QIRYOI; Das *et al.*, 2018 ▸). In this context we have reported the first Co(NCS)_2_ compound, which consists of chains, in which an alternating five- and sixfold Co^II^ coordination is observed (Böhme *et al.*, 2022 ▸).

However, in most of our recent investigations we used pyridine derivatives as coligands, but recently we reported some compounds where we used pyridine *N*-oxide derivatives as coligands (Näther & Jess, 2023 ▸, 2024*a* ▸,*b* ▸). In the course of these investigations we obtained two different discrete complexes by the reaction of Co(NCS)_2_ and 4-methyl­pyridine *N*-oxide (Näther & Jess, 2024*a* ▸). In one of these complexes a trigonal–bipyramidal coordination and in the second complex an octa­hedral coordination is observed, which is surprising because Co(NCS)_2_ compounds with a fivefold coordination and pyridine *N*-oxide coligands were unknown at this time. In further work we used 2-methyl­pyridine *N*-oxide as a coligand, which lead to the formation of Co(NCS)_2_(2-methyl­pyridine *N*-oxide)_3_ in which the Co^II^ cations shows a trigonal–pyramidal coordination as was the case with 4-methyl­pyridine *N*-oxide as coligand (Näther & Jess, 2024*c* ▸). Many experiments were performed but the corresponding octhaedral complex was not obtained. Based on these findings, we decided to try to prepare the corresponding compounds with Co(NCSe)_2_ and in this context it is noted that no seleno­cyanate coordination compounds with pyridine *N*-oxide derivatives and transition-metal cations are reported in the literature (see *Database survey*). Therefore, CoBr_2_, KNCSe and 2-methyl­pyridine *N*-oxide were reacted, which lead to the formation of two different crystals that were investigated by single-crystal X-ray diffraction.
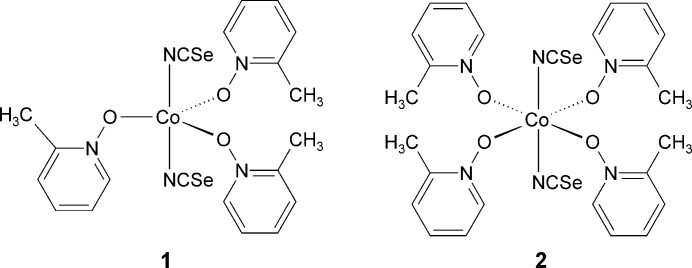


## 2. Structural commentary

**Figure 1:**
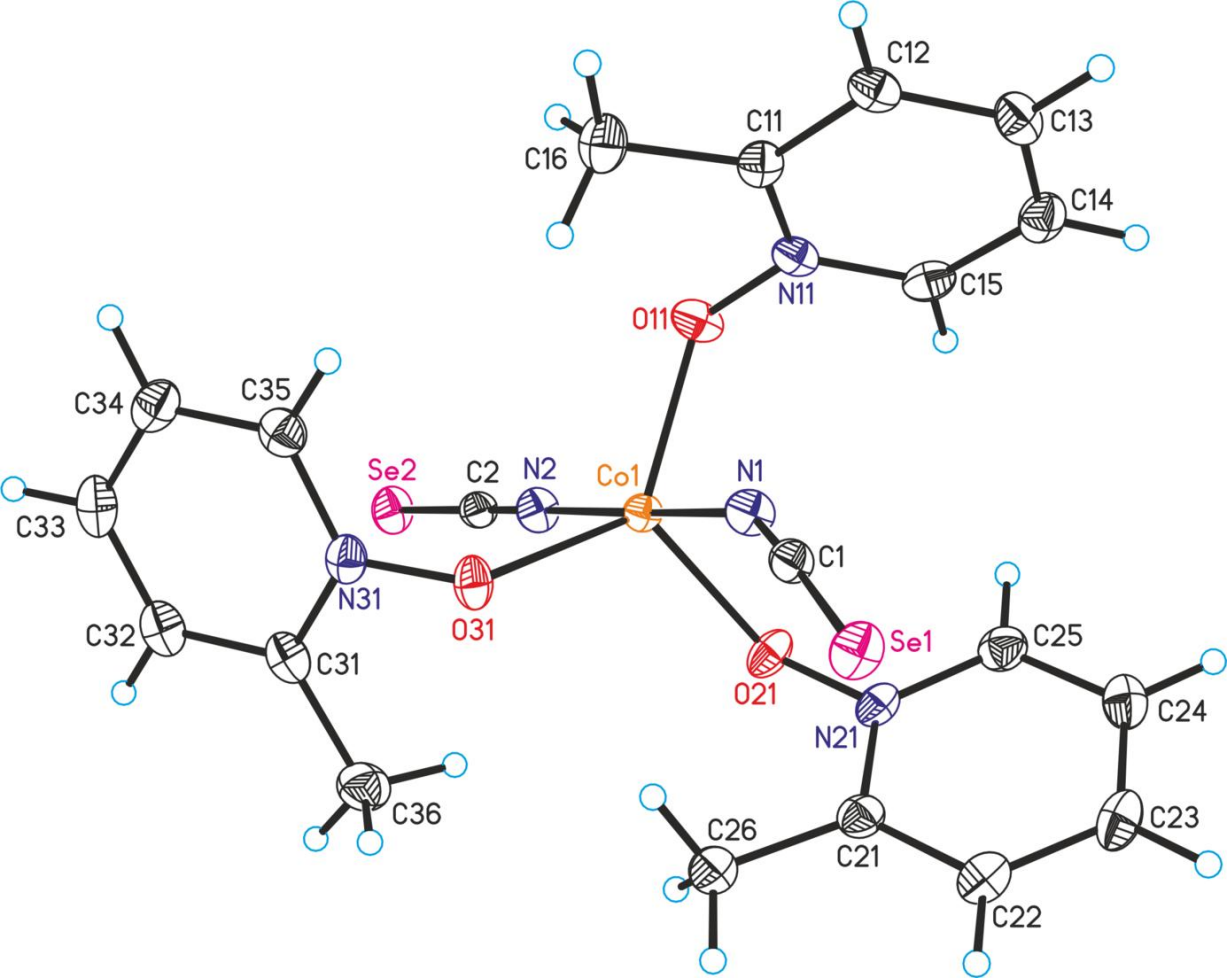
The mol­ecular structure of compound **1** with displacement ellipsoids drawn at the 50% probability level.

The asymmetric unit of compound **1**, Co(NCSe)_2_(C_6_H_7_NO)_3_, consists of one crystallographically independent Co^II^ cation, two independent iso­seleno­cyanate anions and three independent 2-methyl­pyridine *N*-oxide coligands that are located in general positions (Fig. 1 ▸). The metal cations are fivefold coordinated to two terminally N-bonded iso­seleno­cyanate anions and three 2-methyl­pyridine *N*-oxide coligands, forming discrete complexes. The coordination around the metal centers can be described as a slightly distorted trigonal bipyramid with the anionic ligands in the axial and the co­ligands in the equatorial positions (Fig. 1 ▸ and Table 1 ▸). The Co—N bond lengths of the two independent iso­seleno­cyanate anions are significantly different (Table 1 ▸). As expected, the thio­cyanate C—N—Co bond angles are close to linearity, whereas the N—O—Co angles are close to 120°, because only one of the two lone pairs of the oxygen atom is involved in metal coordination (Table 1 ▸). Finally, it is noted that compound **1** is isotypic to the corresponding thio­cyanate complex Co(NCS)_2_(C_6_H_7_NO)_3_, recently reported in the literature (Näther & Jess, 2024*c* ▸).

**Figure 2:**
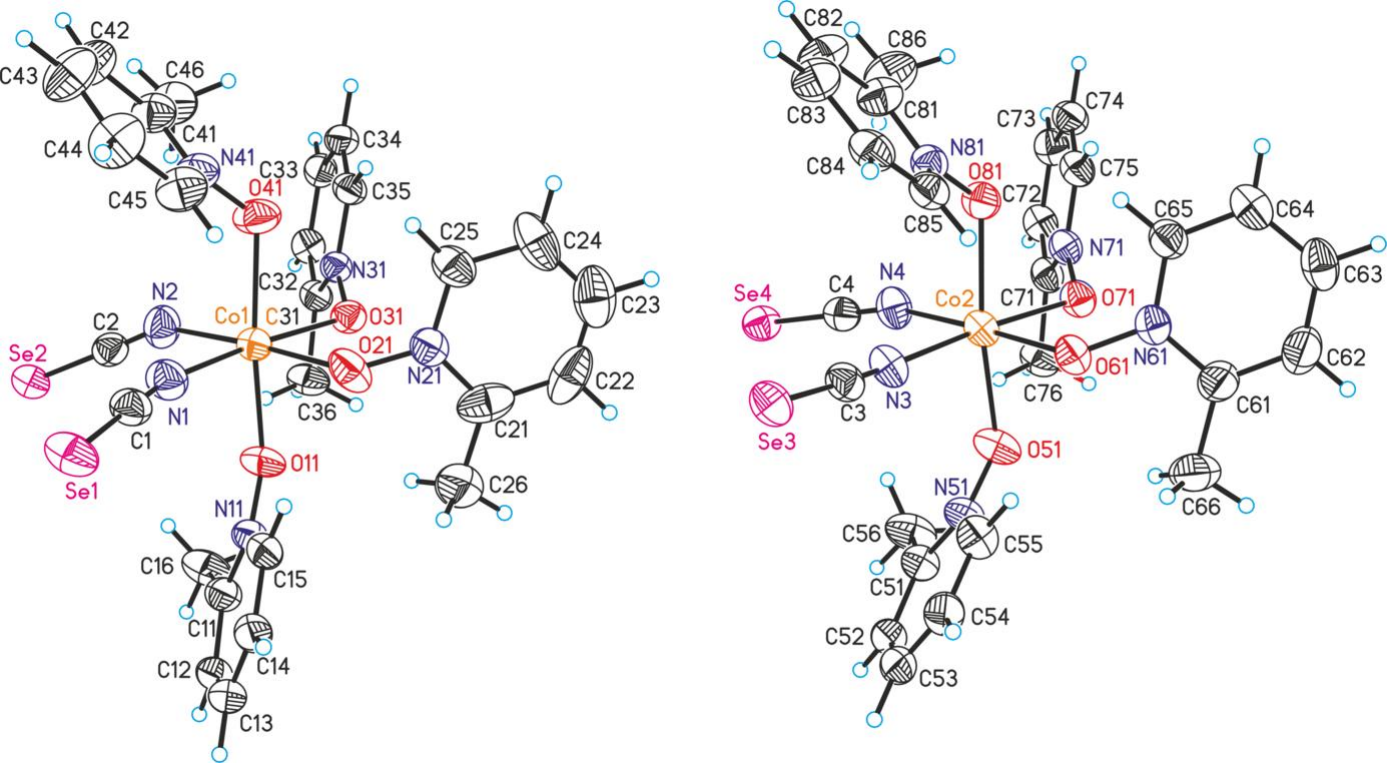
The mol­ecular structure of compound **2** with displacement ellipsoids drawn at the 50% probability level.

In compound **2**, Co(NCS)_2_(C_6_H_7_NO)_4_, two crystallographically independent complexes are present, in which each cobalt cation and each of the two crystallographically independent iso­seleno­cyanate anions and each of the four independent 2-methyl­pyridine *N*-oxide coligands are in general positions (Fig. 2 ▸). In both of the complexes the metal cations are sixfold coordinated to two terminally N-bonded iso­cyanate anions and four 2-methyl­pyridine *N*-oxide ligands within slightly distorted octa­hedra (Fig. 2 ▸ and Table 2 ▸). Bond lengths and angles are comparable in both independent complexes. The Co—N distances of the iso­seleno­cyanate anions and especially of the 2-methyl­pyridine *N*-oxide co­ligands in compound **2** are significantly longer than in compound **1**, which can be traced back to the higher coordination number of the metal ion. As in compound **1**, the C—N—Co angles are more or less linear, whereas the N—O—Co angles tend to be 120° (Table 2 ▸).

## 3. Supra­molecular features

**Figure 3:**
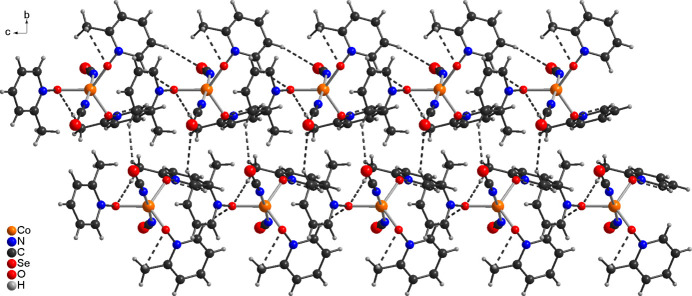
The crystal structure of compound **1** with view of a double chain that propagates along the crystallographic *a*-axis direction: C—H⋯Se and C—H⋯O contacts are shown with dashed lines.

**Figure 4:**
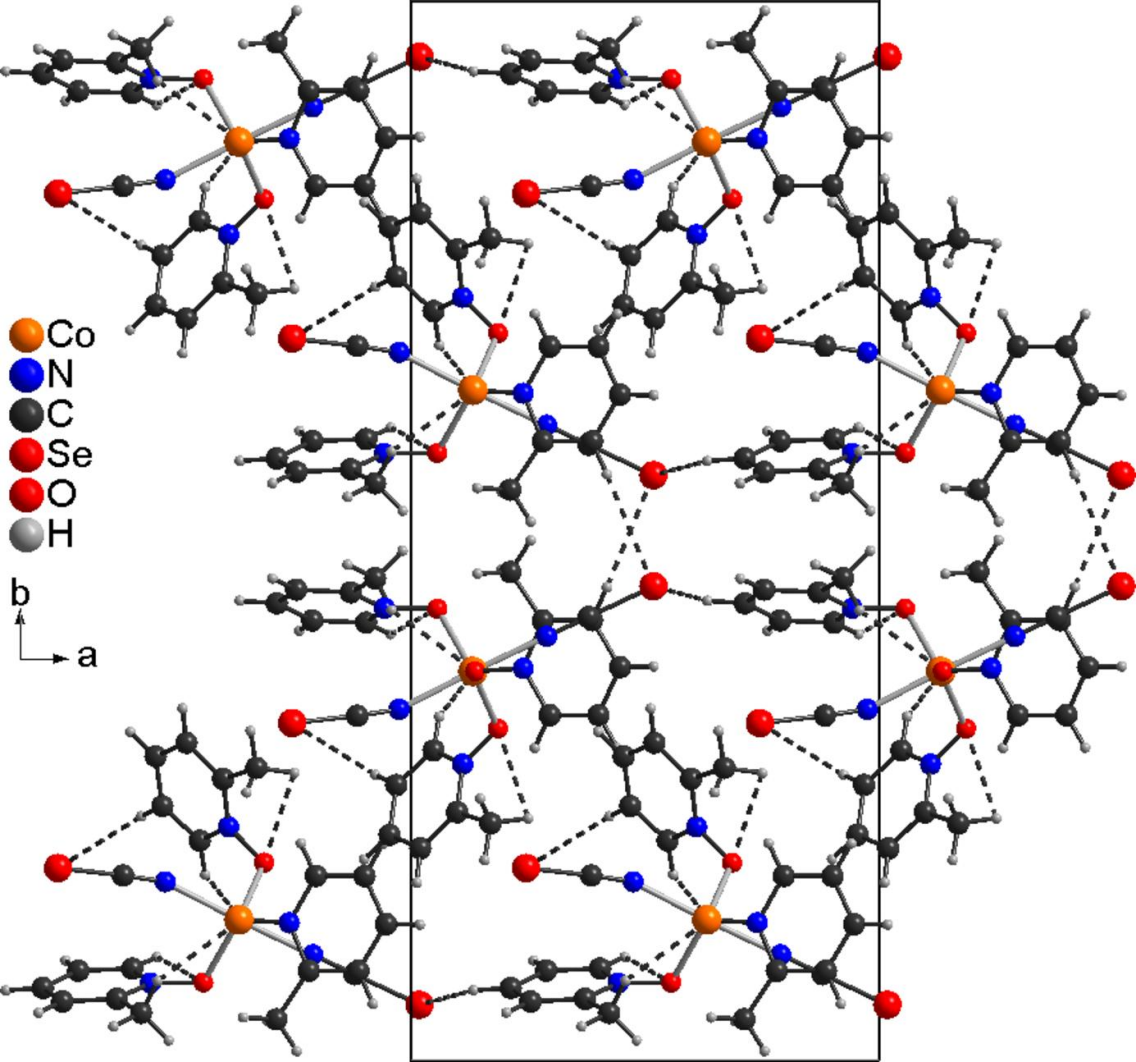
The crystal structure of compound **1** viewed along the crystallographic *c*-axis direction: C—H⋯Se and C—H⋯O contacts are shown with dashed lines.

In the crystal structure of compound **1** the complexes are linked by a relatively short C—H⋯Se contact of 3.00 Å into chains, which are connected into double chains by a slightly longer C—H⋯Se contact of 3.09 Å (Fig. 3 ▸ and Table 3 ▸). Within these chains, weak C—H⋯O contacts are observed (Fig. 3 ▸ and Table 3 ▸). These double chains propagate along the crystallographic *c*-axis direction and no directional inter­molecular inter­actions are observed between them (Fig. 4 ▸).

**Figure 5:**
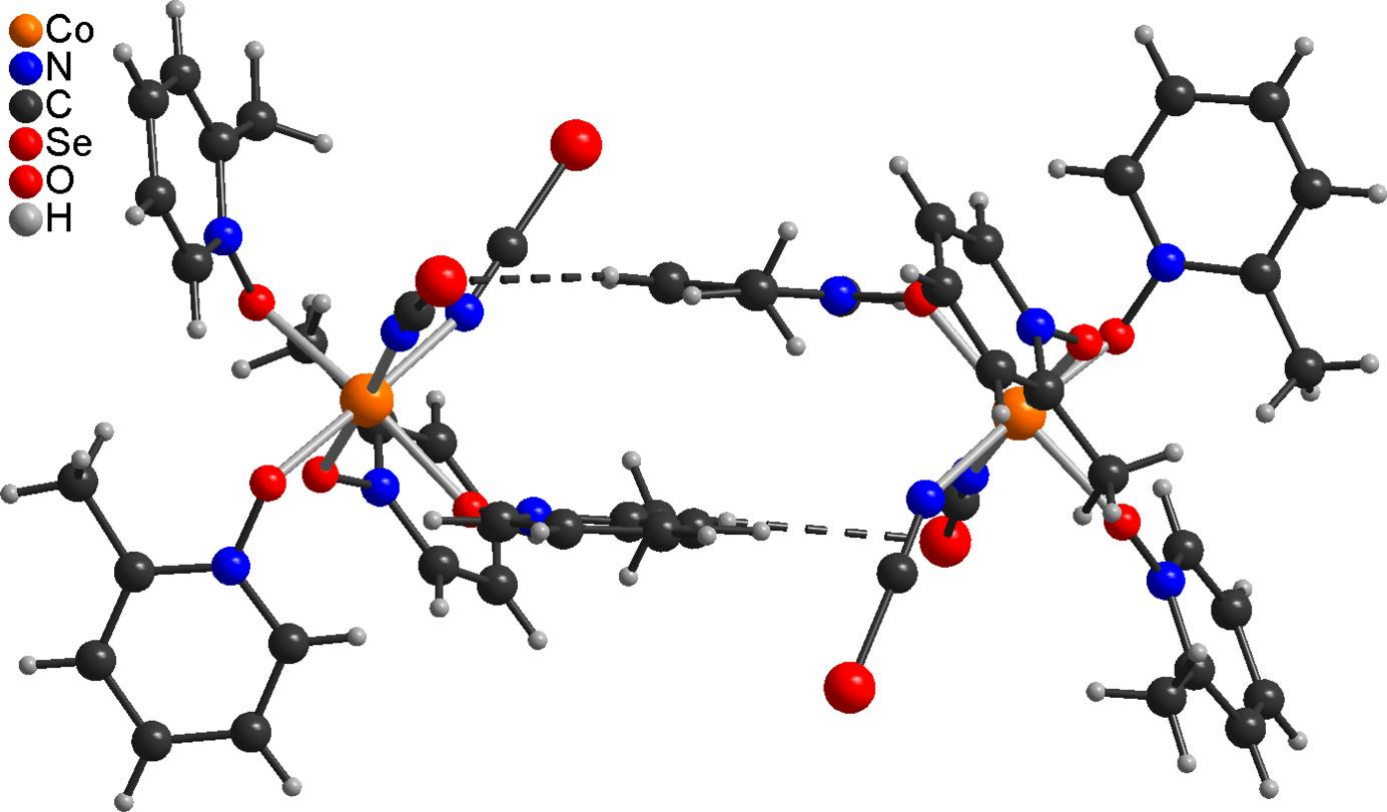
View of the dimeric unit in compound **2** with C—H⋯Se contacts shown with dashed lines.

**Figure 6:**
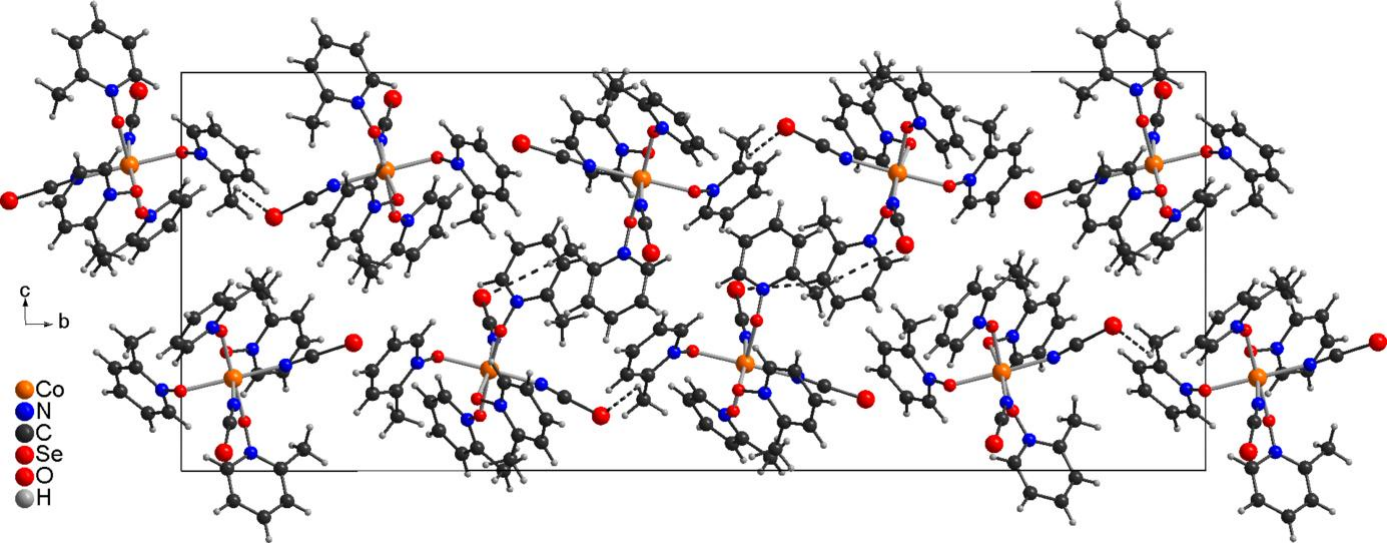
Crystal structure of compound **2** viewed along the crystallographic *a*-axis direction. For clarity only the two C—H⋯Se and C—H⋯O contacts between the dimeric units are shown with dashed lines.

In compound **2** the two crystallographically independent complexes are linked by one relatively short C—H⋯Se contact of 2.87 Å with an C—H⋯Se angle close to linearity into dimeric units (Fig. 5 ▸ and Table 4 ▸). There are numerous additional C—H⋯Se and C—H⋯O contacts with angles far from linearity that connect the dimeric units into a three-dimensional network (Table 4 ▸). Within this network, the complexes the dimeric units are arranged into columns that stack along the crystallographic *a*-axis direction (Fig. 6 ▸).

## 4. Database survey

A search in the CSD (version 5.43, last update March 2023; Groom *et al.*, 2016 ▸) using CONQUEST (Bruno *et al.*, 2002 ▸) reveal that no transition-metal seleno­cyanate coordination compounds with pyridine *N*-oxide derivatives are reported. There is only one compound with 4,4′-bi­pyridine *N*,*N*′-dioxide with the composition Co(NCS)_2_(4,4′-bi­pyridine *N*,*N*′-diox­ide)(H_2_O)_2_·H_2_O, in which the cobalt cations are octa­hedrally coordinated to two seleno­cyanate anions, two water mol­ecules and two O atoms of the 4,4′-bi­pyridine *N*,*N*′-dioxide coligands and are linked into chains by the bridging 4,4′-bi­pyridine *N*,*N*′-dioxide ligands (ROLJEI; Jana *et al.*, 2007 ▸).

It is also noted that with Co(NCS)_2_ and pyridine *N*-oxide derivatives no structures with a fivefold coordination are reported in the CSD. There is only one recent example, which is Co(NCS)_2_(2-methyl­pyridine *N*-oxide)_3_, already mentioned in the *Chemical context* section (Näther & Jess, 2024*c* ▸).

## 5. Synthesis and crystallization

CoBr_2_ (99%), KNCSe (98.5%) and 2-methyl­pyridine *N*-oxide (96%) were purchased from Sigma Aldrich. 0.25 mmol (54.7 mg) of CoBr_2_, 0.5 mmol of KNCSe (72.0 mg) and 4 mmol (436.4 mg) of 2-methyl­pyridine *N*-oxide in 1 ml of ethanol were reacted for 3 d at room temperature, which led to the formation of crystals of compound **1** (pink needles) and **2** (pink blocks) in the same batch. Reacting the components in the stoichiometric ratios given by the formulae were also tried but pure crystalline phases were never obtained: powder X-ray diffraction shows that they are always contaminated with additional unknown crystalline phases and with KBr, which is also a product of this reaction.

## 6. Refinement

Crystal data, data collection and structure refinement details are summarized in Table 5 ▸. The hydrogen atoms were positioned with idealized geometry (methyl H atoms allowed to rotate and not to tip) and were refined with *U*_ιso_(H) = 1.2*U*_eq_(C) (1.5 for methyl H atoms) using a riding model.

## Supplementary Material

Crystal structure: contains datablock(s) global, 2, 1. DOI: 10.1107/S2056989024005073/hb8098sup1.cif

Structure factors: contains datablock(s) 1. DOI: 10.1107/S2056989024005073/hb80981sup2.hkl

Structure factors: contains datablock(s) 2. DOI: 10.1107/S2056989024005073/hb80982sup3.hkl

CCDC references: 2359034, 2359033

Additional supporting information:  crystallographic information; 3D view; checkCIF report

## Figures and Tables

**Table 1:** Selected geometric parameters (Å, °) for **1**[Chem scheme1]

Co1—N1	2.090 (3)	Co1—O21	2.011 (2)
Co1—N2	2.047 (3)	Co1—O31	2.032 (2)
Co1—O11	1.989 (2)		
			
N2—Co1—N1	176.24 (11)	O31—Co1—N1	84.07 (10)
O11—Co1—N1	91.77 (10)	O31—Co1—N2	92.72 (10)
O11—Co1—N2	88.63 (10)	C1—N1—Co1	156.2 (3)
O11—Co1—O21	115.08 (11)	C2—N2—Co1	177.2 (2)
O11—Co1—O31	127.68 (10)	N11—O11—Co1	118.91 (18)
O21—Co1—N1	91.29 (10)	N21—O21—Co1	120.92 (18)
O21—Co1—N2	91.92 (10)	N31—O31—Co1	118.01 (18)
O21—Co1—O31	117.13 (10)		

**Table 2:** Selected geometric parameters (Å, °) for **2**[Chem scheme1]

Co1—N1	2.105 (3)	Co2—N3	2.096 (3)
Co1—N2	2.096 (3)	Co2—N4	2.115 (3)
Co1—O11	2.104 (2)	Co2—O51	2.092 (2)
Co1—O21	2.081 (2)	Co2—O61	2.109 (2)
Co1—O31	2.103 (2)	Co2—O71	2.102 (2)
Co1—O41	2.086 (2)	Co2—O81	2.098 (2)
			
N2—Co1—N1	92.43 (11)	N3—Co2—N4	92.80 (10)
N2—Co1—O11	85.49 (11)	N3—Co2—O61	85.67 (9)
N2—Co1—O31	95.84 (10)	N3—Co2—O71	173.62 (9)
O11—Co1—N1	94.63 (10)	N3—Co2—O81	95.39 (9)
O21—Co1—N1	86.10 (10)	O51—Co2—N3	94.08 (10)
O21—Co1—N2	176.62 (11)	O51—Co2—N4	88.66 (10)
O21—Co1—O11	91.58 (10)	O51—Co2—O61	90.02 (9)
O21—Co1—O31	85.57 (8)	O51—Co2—O71	84.49 (9)
O21—Co1—O41	92.05 (10)	O51—Co2—O81	170.51 (9)
O31—Co1—N1	171.62 (10)	O61—Co2—N4	177.90 (9)
O31—Co1—O11	84.73 (8)	O71—Co2—N4	93.37 (9)
O41—Co1—N1	96.79 (10)	O71—Co2—O61	88.12 (8)
O41—Co1—N2	91.15 (12)	O81—Co2—N4	91.46 (10)
O41—Co1—O11	168.23 (9)	O81—Co2—O61	90.12 (8)
O41—Co1—O31	84.39 (8)	O81—Co2—O71	86.03 (8)
C1—N1—Co1	166.2 (3)	C3—N3—Co2	167.6 (2)
C2—N2—Co1	151.9 (3)	C4—N4—Co2	163.8 (3)
N11—O11—Co1	128.93 (17)	N51—O51—Co2	123.32 (18)
N21—O21—Co1	121.76 (17)	N61—O61—Co2	123.03 (16)
N31—O31—Co1	120.96 (15)	N71—O71—Co2	120.08 (16)
N41—O41—Co1	124.68 (19)	N81—O81—Co2	124.24 (16)

**Table 3:** Hydrogen-bond geometry (Å, °) for **1**[Chem scheme1]

*D*—H⋯*A*	*D*—H	H⋯*A*	*D*⋯*A*	*D*—H⋯*A*
C14—H14⋯Se1^i^	0.95	3.00	3.920 (3)	163
C15—H15⋯O31^i^	0.95	2.49	3.305 (4)	144
C32—H32⋯Se2^ii^	0.95	3.09	3.945 (4)	151
C26—H26*B*⋯O31	0.98	2.62	3.511 (4)	151

Symmetry codes: (i) 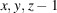; (ii) 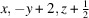.

**Table 4:** Hydrogen-bond geometry (Å, °) for **2**[Chem scheme1]

*D*—H⋯*A*	*D*—H	H⋯*A*	*D*⋯*A*	*D*—H⋯*A*
C15—H15⋯O21	0.95	2.36	3.158 (4)	141
C26—H26*C*⋯O11	0.98	2.49	3.367 (5)	149
C35—H35⋯Se1^i^	0.95	2.96	3.748 (3)	141
C36—H36*C*⋯O11	0.98	2.55	3.281 (4)	131
C42—H42⋯Se3^ii^	0.95	2.87	3.811 (4)	171
C52—H52⋯Se2^iii^	0.95	3.05	3.926 (3)	153
C55—H55⋯O61	0.95	2.45	3.245 (4)	141
C56—H56*B*⋯Se2^iii^	0.98	3.02	3.933 (3)	156
C65—H65⋯O81	0.95	2.40	3.140 (4)	135
C66—H66*A*⋯Se2^iv^	0.98	3.14	3.807 (5)	126
C75—H75⋯Se3^i^	0.95	2.95	3.726 (3)	140
C75—H75⋯O81	0.95	2.62	3.075 (3)	110
C76—H76*C*⋯O51	0.98	2.61	3.354 (4)	133
C86—H86*B*⋯Se1^ii^	0.98	3.14	4.109 (4)	169

Symmetry codes: (i) 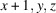; (ii) 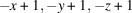; (iii) 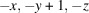; (iv) 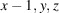.

**Table 5:** Experimental details

	**1**	**2**
Crystal data		
Chemical formula	[Co(NCSe)_2_(C_6_H_7_NO)_3_]	[Co(NCSe)_2_(C_6_H_7_NO)_4_]
*M*_r_	596.27	705.39
Crystal system, space group	Monoclinic, *Cc*	Monoclinic, *P*2_1_/*n*
Temperature (K)	100	100
*a*, *b*, *c* (Å)	12.07686 (6), 26.47661 (16), 7.25623 (4)	9.3825 (1), 39.9164 (3), 15.9920 (1)
β (°)	104.2765 (5)	104.217 (1)
*V* (Å^3^)	2248.56 (2)	5805.82 (9)
*Z*	4	8
Radiation type	Cu *K*α	Cu *K*α
μ (mm^−1^)	9.96	7.86
Crystal size (mm)	0.18 × 0.06 × 0.04	0.16 × 0.14 × 0.12

Data collection		
Diffractometer	XtaLAB Synergy, Dualflex, HyPix	XtaLAB Synergy, Dualflex, HyPix
Absorption correction	Multi-scan (CrysAlisPr; Rigaku OD, 2023 ▸)	Multi-scan (*CrysAlis PRO*; Rigaku OD, 2023 ▸)
*T*_min_, *T*_max_	0.468, 1.000	0.489, 0.596
No. of measured, independent and observed [*I* > 2σ(*I*)] reflections	49619, 4828, 4788	41815, 11937, 11197
*R*_int_	0.035	0.020
(sin θ/λ)_max_ (Å^−1^)	0.640	0.640

Refinement		
*R*[*F*^2^ > 2σ(*F*^2^)], *wR*(*F*^2^), *S*	0.020, 0.062, 0.83	0.038, 0.109, 1.05
No. of reflections	4828	11937
No. of parameters	283	711
No. of restraints	2	0
H-atom treatment	H-atom parameters constrained	H-atom parameters constrained
Δρ_max_, Δρ_min_ (e Å^−3^)	0.25, −0.40	1.76, −0.67
Absolute structure	Flack *x* determined using 2302 quotients [(*I*^+^)−(*I*^−^)]/[(*I*^+^)+(*I*^−^)] (Parsons *et al.*, 2013 ▸)	–
Absolute structure parameter	−0.0305 (14)	–

Computer programs: *CrysAlis PRO* (Rigaku OD, 2023 ▸), *SHELXT* (Sheldrick, 2015*a* ▸), *SHELXL2016/6* (Sheldrick, 2015*b* ▸), *DIAMOND* (Brandenburg & Putz, 1999 ▸); *XP* in *SHELXTL-PC* (Sheldrick, 2008 ▸), *OLEX2* (Dolomanov *et al.*, 2009 ▸) and *publCIF* (Westrip, 2010 ▸).

## supplementary crystallographic information

### Diisoselenocyanatotris(2-methylpyridine N-oxide)cobalt(II) (1) Crystal data

**Table:**

[Co(NCSe)_2_(C_6_H_7_NO)_3_]	*F*(000) = 1180
*M**_r_* = 596.27	*D*_x_ = 1.761 Mg m^−^^3^
Monoclinic, *C**c*	Cu *K*α radiation, λ = 1.54184 Å
*a* = 12.07686 (6) Å	Cell parameters from 35276 reflections
*b* = 26.47661 (16) Å	θ = 3.3–79.5°
*c* = 7.25623 (4) Å	µ = 9.96 mm^−^^1^
β = 104.2765 (5)°	*T* = 100 K
*V* = 2248.56 (2) Å^3^	Needle, pink
*Z* = 4	0.18 × 0.06 × 0.04 mm

### Diisoselenocyanatotris(2-methylpyridine N-oxide)cobalt(II) (1) Data collection

**Table:**

XtaLAB Synergy, Dualflex, HyPix diffractometer	4828 independent reflections
Radiation source: micro-focus sealed X-ray tube, PhotonJet (Cu) X-ray Source	4788 reflections with *I* > 2σ(*I*)
Mirror monochromator	*R*_int_ = 0.035
Detector resolution: 10.0000 pixels mm^-1^	θ_max_ = 80.4°, θ_min_ = 3.3°
ω scans	*h* = −15→15
Absorption correction: multi-scan (CrysAlisPr; Rigaku OD, 2023)	*k* = −29→33
*T*_min_ = 0.468, *T*_max_ = 1.000	*l* = −9→9
49619 measured reflections	

### Diisoselenocyanatotris(2-methylpyridine N-oxide)cobalt(II) (1) Refinement

**Table:**

Refinement on *F*^2^	Hydrogen site location: inferred from neighbouring sites
Least-squares matrix: full	H-atom parameters constrained
*R*[*F*^2^ > 2σ(*F*^2^)] = 0.020	*w* = 1/[σ^2^(*F*_o_^2^) + (0.0682*P*)^2^ + 0.5399*P*] where *P* = (*F*_o_^2^ + 2*F*_c_^2^)/3
*wR*(*F*^2^) = 0.062	(Δ/σ)_max_ = 0.001
*S* = 0.83	Δρ_max_ = 0.25 e Å^−^^3^
4828 reflections	Δρ_min_ = −0.40 e Å^−^^3^
283 parameters	Absolute structure: Flack *x* determined using 2302 quotients [(*I*^+^)-(*I*^-^)]/[(*I*^+^)+(*I*^-^)] (Parsons *et al.*, 2013)
2 restraints	Absolute structure parameter: −0.0305 (14)
Primary atom site location: dual	

### Diisoselenocyanatotris(2-methylpyridine N-oxide)cobalt(II) (1) Special details

**Table:**

Geometry. All esds (except the esd in the dihedral angle between two l.s. planes) are estimated using the full covariance matrix. The cell esds are taken into account individually in the estimation of esds in distances, angles and torsion angles; correlations between esds in cell parameters are only used when they are defined by crystal symmetry. An approximate (isotropic) treatment of cell esds is used for estimating esds involving l.s. planes.

### Diisoselenocyanatotris(2-methylpyridine N-oxide)cobalt(II) (1) Fractional atomic coordinates and isotropic or equivalent isotropic displacement parameters (Å^2^)

**Table:**

	*x*	*y*	*z*	*U*_iso_*/*U*_eq_	
Co1	0.63215 (4)	0.86731 (2)	0.79899 (6)	0.01397 (11)	
N1	0.4748 (2)	0.83139 (10)	0.7711 (4)	0.0180 (5)	
C1	0.3847 (3)	0.82543 (12)	0.7960 (4)	0.0173 (6)	
Se1	0.24577 (3)	0.81838 (2)	0.84143 (4)	0.02001 (9)	
N2	0.7899 (2)	0.90028 (10)	0.8433 (4)	0.0184 (5)	
C2	0.8794 (3)	0.91870 (12)	0.8762 (4)	0.0172 (6)	
Se2	1.01804 (3)	0.94751 (2)	0.92765 (4)	0.02230 (10)	
O11	0.6860 (2)	0.81331 (8)	0.6514 (4)	0.0209 (5)	
N11	0.6102 (2)	0.77961 (10)	0.5563 (4)	0.0169 (5)	
C11	0.5986 (3)	0.73433 (12)	0.6337 (4)	0.0173 (6)	
C12	0.5201 (3)	0.70021 (12)	0.5273 (4)	0.0185 (6)	
H12	0.512002	0.667709	0.577882	0.022*	
C13	0.4543 (3)	0.71294 (13)	0.3504 (5)	0.0210 (6)	
H13	0.399759	0.689803	0.280355	0.025*	
C14	0.4686 (3)	0.76001 (13)	0.2754 (5)	0.0218 (6)	
H14	0.423837	0.769563	0.153540	0.026*	
C15	0.5483 (3)	0.79260 (12)	0.3801 (5)	0.0204 (6)	
H15	0.560187	0.824548	0.328556	0.024*	
O21	0.55635 (18)	0.92647 (9)	0.6444 (3)	0.0199 (4)	
N21	0.4429 (2)	0.92698 (10)	0.5685 (4)	0.0178 (5)	
C21	0.3714 (3)	0.94192 (11)	0.6761 (5)	0.0194 (6)	
C22	0.2550 (3)	0.94443 (12)	0.5905 (5)	0.0220 (6)	
H22	0.203471	0.955060	0.662840	0.026*	
C23	0.2133 (3)	0.93161 (15)	0.4009 (5)	0.0247 (7)	
H23	0.133828	0.933923	0.342723	0.030*	
C24	0.2889 (3)	0.91531 (13)	0.2967 (5)	0.0236 (7)	
H24	0.261750	0.905660	0.167347	0.028*	
C25	0.4040 (3)	0.91336 (12)	0.3842 (5)	0.0219 (6)	
H25	0.456510	0.902355	0.314261	0.026*	
O31	0.6368 (2)	0.86742 (9)	1.0809 (3)	0.0189 (4)	
N31	0.7399 (2)	0.87014 (10)	1.2061 (4)	0.0183 (5)	
C31	0.7818 (3)	0.91535 (13)	1.2802 (4)	0.0204 (6)	
C32	0.8872 (3)	0.91558 (14)	1.4123 (5)	0.0237 (7)	
H32	0.918537	0.946696	1.466300	0.028*	
C33	0.9469 (3)	0.87135 (15)	1.4660 (5)	0.0250 (7)	
H33	1.018835	0.871934	1.556260	0.030*	
C34	0.9007 (3)	0.82600 (14)	1.3865 (5)	0.0237 (7)	
H34	0.940708	0.795231	1.421995	0.028*	
C35	0.7968 (3)	0.82592 (13)	1.2565 (5)	0.0207 (6)	
H35	0.764512	0.795027	1.201666	0.025*	
C36	0.7123 (3)	0.96119 (14)	1.2166 (5)	0.0272 (7)	
H36A	0.641642	0.959482	1.259800	0.041*	
H36B	0.693531	0.963154	1.077488	0.041*	
H36C	0.755827	0.991225	1.270547	0.041*	
C16	0.6698 (3)	0.72386 (14)	0.8287 (5)	0.0254 (7)	
H16A	0.648341	0.747113	0.919137	0.038*	
H16B	0.750650	0.728605	0.831326	0.038*	
H16C	0.657240	0.688988	0.864016	0.038*	
C26	0.4234 (3)	0.95514 (13)	0.8779 (5)	0.0240 (7)	
H26A	0.481990	0.981121	0.883718	0.036*	
H26B	0.458476	0.924982	0.946474	0.036*	
H26C	0.364160	0.968028	0.936542	0.036*	

### Diisoselenocyanatotris(2-methylpyridine N-oxide)cobalt(II) (1) Atomic displacement parameters (Å^2^)

**Table:**

	*U*^11^	*U*^22^	*U*^33^	*U*^12^	*U*^13^	*U*^23^
Co1	0.0123 (2)	0.0146 (2)	0.0155 (2)	−0.00053 (16)	0.00419 (17)	−0.00040 (16)
N1	0.0142 (12)	0.0190 (12)	0.0223 (12)	−0.0031 (10)	0.0076 (10)	−0.0020 (10)
C1	0.0202 (16)	0.0154 (13)	0.0156 (13)	0.0006 (11)	0.0033 (11)	−0.0004 (10)
Se1	0.01337 (15)	0.02559 (18)	0.02267 (16)	−0.00155 (12)	0.00749 (11)	0.00275 (12)
N2	0.0150 (11)	0.0198 (13)	0.0207 (12)	−0.0042 (9)	0.0052 (9)	−0.0003 (10)
C2	0.0212 (16)	0.0171 (14)	0.0141 (13)	0.0035 (11)	0.0058 (11)	0.0010 (10)
Se2	0.01459 (16)	0.02685 (19)	0.02437 (17)	−0.00539 (12)	0.00272 (12)	−0.00185 (12)
O11	0.0140 (11)	0.0206 (11)	0.0298 (12)	−0.0048 (8)	0.0087 (9)	−0.0088 (9)
N11	0.0111 (11)	0.0182 (13)	0.0229 (12)	−0.0018 (9)	0.0073 (9)	−0.0058 (10)
C11	0.0135 (13)	0.0216 (15)	0.0184 (13)	0.0005 (11)	0.0069 (11)	0.0000 (11)
C12	0.0170 (14)	0.0193 (15)	0.0221 (14)	−0.0012 (11)	0.0102 (11)	−0.0036 (12)
C13	0.0143 (14)	0.0268 (16)	0.0228 (15)	−0.0042 (12)	0.0064 (11)	−0.0062 (12)
C14	0.0169 (14)	0.0283 (17)	0.0210 (14)	0.0041 (12)	0.0061 (11)	0.0007 (12)
C15	0.0202 (14)	0.0180 (14)	0.0256 (15)	0.0042 (12)	0.0107 (12)	0.0025 (12)
O21	0.0115 (10)	0.0198 (11)	0.0272 (11)	−0.0002 (8)	0.0026 (8)	0.0065 (9)
N21	0.0153 (12)	0.0161 (12)	0.0215 (12)	0.0014 (9)	0.0035 (10)	0.0044 (10)
C21	0.0219 (16)	0.0151 (14)	0.0211 (15)	0.0015 (11)	0.0050 (13)	0.0021 (11)
C22	0.0188 (16)	0.0219 (16)	0.0264 (16)	0.0005 (11)	0.0075 (13)	0.0056 (11)
C23	0.0162 (14)	0.0279 (17)	0.0272 (16)	−0.0020 (13)	0.0001 (12)	0.0065 (13)
C24	0.0243 (16)	0.0250 (17)	0.0198 (14)	−0.0025 (13)	0.0021 (12)	0.0012 (12)
C25	0.0244 (15)	0.0221 (15)	0.0204 (14)	0.0045 (13)	0.0076 (12)	0.0034 (12)
O31	0.0143 (10)	0.0262 (12)	0.0154 (10)	−0.0044 (8)	0.0018 (8)	0.0000 (8)
N31	0.0160 (12)	0.0242 (14)	0.0153 (11)	−0.0008 (10)	0.0048 (10)	0.0015 (9)
C31	0.0212 (15)	0.0237 (16)	0.0174 (14)	−0.0062 (12)	0.0067 (12)	−0.0021 (11)
C32	0.0224 (16)	0.0298 (17)	0.0195 (14)	−0.0063 (12)	0.0061 (12)	−0.0021 (12)
C33	0.0192 (15)	0.038 (2)	0.0167 (14)	−0.0049 (13)	0.0031 (12)	0.0019 (13)
C34	0.0199 (15)	0.0302 (17)	0.0225 (15)	0.0053 (13)	0.0082 (12)	0.0067 (13)
C35	0.0235 (16)	0.0216 (15)	0.0187 (14)	−0.0017 (12)	0.0082 (12)	0.0004 (11)
C36	0.0259 (17)	0.0233 (17)	0.0313 (17)	−0.0018 (14)	0.0050 (14)	−0.0060 (14)
C16	0.0202 (15)	0.0311 (18)	0.0235 (16)	−0.0011 (12)	0.0029 (12)	0.0015 (13)
C26	0.0269 (18)	0.0223 (15)	0.0217 (15)	0.0038 (13)	0.0041 (13)	0.0004 (12)

### Diisoselenocyanatotris(2-methylpyridine N-oxide)cobalt(II) (1) Geometric parameters (Å, º)

**Table:**

Co1—N1	2.090 (3)	C22—C23	1.385 (5)
Co1—N2	2.047 (3)	C23—H23	0.9500
Co1—O11	1.989 (2)	C23—C24	1.390 (5)
Co1—O21	2.011 (2)	C24—H24	0.9500
Co1—O31	2.032 (2)	C24—C25	1.379 (5)
N1—C1	1.157 (4)	C25—H25	0.9500
C1—Se1	1.797 (3)	O31—N31	1.350 (4)
N2—C2	1.156 (4)	N31—C31	1.358 (4)
C2—Se2	1.794 (3)	N31—C35	1.361 (4)
O11—N11	1.341 (3)	C31—C32	1.392 (5)
N11—C11	1.346 (4)	C31—C36	1.483 (5)
N11—C15	1.357 (4)	C32—H32	0.9500
C11—C12	1.397 (4)	C32—C33	1.380 (5)
C11—C16	1.490 (4)	C33—H33	0.9500
C12—H12	0.9500	C33—C34	1.388 (5)
C12—C13	1.374 (5)	C34—H34	0.9500
C13—H13	0.9500	C34—C35	1.371 (5)
C13—C14	1.387 (5)	C35—H35	0.9500
C14—H14	0.9500	C36—H36A	0.9800
C14—C15	1.374 (5)	C36—H36B	0.9800
C15—H15	0.9500	C36—H36C	0.9800
O21—N21	1.345 (3)	C16—H16A	0.9800
N21—C21	1.358 (4)	C16—H16B	0.9800
N21—C25	1.353 (4)	C16—H16C	0.9800
C21—C22	1.392 (5)	C26—H26A	0.9800
C21—C26	1.485 (4)	C26—H26B	0.9800
C22—H22	0.9500	C26—H26C	0.9800
			
N2—Co1—N1	176.24 (11)	C24—C23—H23	120.4
O11—Co1—N1	91.77 (10)	C23—C24—H24	120.5
O11—Co1—N2	88.63 (10)	C25—C24—C23	119.0 (3)
O11—Co1—O21	115.08 (11)	C25—C24—H24	120.5
O11—Co1—O31	127.68 (10)	N21—C25—C24	120.6 (3)
O21—Co1—N1	91.29 (10)	N21—C25—H25	119.7
O21—Co1—N2	91.92 (10)	C24—C25—H25	119.7
O21—Co1—O31	117.13 (10)	N31—O31—Co1	118.01 (18)
O31—Co1—N1	84.07 (10)	O31—N31—C31	120.3 (3)
O31—Co1—N2	92.72 (10)	O31—N31—C35	117.1 (3)
C1—N1—Co1	156.2 (3)	C31—N31—C35	122.5 (3)
N1—C1—Se1	177.6 (3)	N31—C31—C32	117.7 (3)
C2—N2—Co1	177.2 (2)	N31—C31—C36	118.1 (3)
N2—C2—Se2	179.8 (3)	C32—C31—C36	124.2 (3)
N11—O11—Co1	118.91 (18)	C31—C32—H32	119.5
O11—N11—C11	120.4 (3)	C33—C32—C31	121.1 (3)
O11—N11—C15	117.6 (3)	C33—C32—H32	119.5
C11—N11—C15	121.9 (3)	C32—C33—H33	120.4
N11—C11—C12	118.2 (3)	C32—C33—C34	119.2 (3)
N11—C11—C16	117.8 (3)	C34—C33—H33	120.4
C12—C11—C16	124.0 (3)	C33—C34—H34	120.2
C11—C12—H12	119.5	C35—C34—C33	119.6 (3)
C13—C12—C11	121.0 (3)	C35—C34—H34	120.2
C13—C12—H12	119.5	N31—C35—C34	119.9 (3)
C12—C13—H13	120.4	N31—C35—H35	120.0
C12—C13—C14	119.2 (3)	C34—C35—H35	120.0
C14—C13—H13	120.4	C31—C36—H36A	109.5
C13—C14—H14	120.5	C31—C36—H36B	109.5
C15—C14—C13	119.0 (3)	C31—C36—H36C	109.5
C15—C14—H14	120.5	H36A—C36—H36B	109.5
N11—C15—C14	120.7 (3)	H36A—C36—H36C	109.5
N11—C15—H15	119.7	H36B—C36—H36C	109.5
C14—C15—H15	119.7	C11—C16—H16A	109.5
N21—O21—Co1	120.92 (18)	C11—C16—H16B	109.5
O21—N21—C21	119.7 (3)	C11—C16—H16C	109.5
O21—N21—C25	118.2 (3)	H16A—C16—H16B	109.5
C21—N21—C25	122.1 (3)	H16A—C16—H16C	109.5
N21—C21—C22	118.2 (3)	H16B—C16—H16C	109.5
N21—C21—C26	117.5 (3)	C21—C26—H26A	109.5
C22—C21—C26	124.3 (3)	C21—C26—H26B	109.5
C21—C22—H22	119.6	C21—C26—H26C	109.5
C23—C22—C21	120.8 (3)	H26A—C26—H26B	109.5
C23—C22—H22	119.6	H26A—C26—H26C	109.5
C22—C23—H23	120.4	H26B—C26—H26C	109.5
C22—C23—C24	119.3 (3)		

### Diisoselenocyanatotris(2-methylpyridine N-oxide)cobalt(II) (1) Hydrogen-bond geometry (Å, º)

**Table:**

*D*—H···*A*	*D*—H	H···*A*	*D*···*A*	*D*—H···*A*
C14—H14···Se1^i^	0.95	3.00	3.920 (3)	163
C15—H15···O31^i^	0.95	2.49	3.305 (4)	144
C32—H32···Se2^ii^	0.95	3.09	3.945 (4)	151
C26—H26*B*···O31	0.98	2.62	3.511 (4)	151

Symmetry codes: (i) *x*, *y*, *z*−1; (ii) *x*, −*y*+2, *z*+1/2.

### Diisoselenocyanatotetrakis(2-methylpyridine N-oxide)cobalt(II) (2) Crystal data

**Table:**

[Co(NCSe)_2_(C_6_H_7_NO)_4_]	*F*(000) = 2824
*M**_r_* = 705.39	*D*_x_ = 1.614 Mg m^−^^3^
Monoclinic, *P*2_1_/*n*	Cu *K*α radiation, λ = 1.54184 Å
*a* = 9.3825 (1) Å	Cell parameters from 27892 reflections
*b* = 39.9164 (3) Å	θ = 3.1–79.1°
*c* = 15.9920 (1) Å	µ = 7.86 mm^−^^1^
β = 104.217 (1)°	*T* = 100 K
*V* = 5805.82 (9) Å^3^	Block, pink
*Z* = 8	0.16 × 0.14 × 0.12 mm

### Diisoselenocyanatotetrakis(2-methylpyridine N-oxide)cobalt(II) (2) Data collection

**Table:**

XtaLAB Synergy, Dualflex, HyPix diffractometer	11937 independent reflections
Radiation source: micro-focus sealed X-ray tube, PhotonJet (Cu) X-ray Source	11197 reflections with *I* > 2σ(*I*)
Mirror monochromator	*R*_int_ = 0.020
Detector resolution: 10.0000 pixels mm^-1^	θ_max_ = 80.4°, θ_min_ = 2.2°
ω scans	*h* = −7→11
Absorption correction: multi-scan (CrysAlisPro; Rigaku OD, 2023)	*k* = −49→50
*T*_min_ = 0.489, *T*_max_ = 0.596	*l* = −20→20
41815 measured reflections	

### Diisoselenocyanatotetrakis(2-methylpyridine N-oxide)cobalt(II) (2) Refinement

**Table:**

Refinement on *F*^2^	Primary atom site location: dual
Least-squares matrix: full	Hydrogen site location: inferred from neighbouring sites
*R*[*F*^2^ > 2σ(*F*^2^)] = 0.038	H-atom parameters constrained
*wR*(*F*^2^) = 0.109	*w* = 1/[σ^2^(*F*_o_^2^) + (0.0611*P*)^2^ + 6.3002*P*] where *P* = (*F*_o_^2^ + 2*F*_c_^2^)/3
*S* = 1.05	(Δ/σ)_max_ = 0.003
11937 reflections	Δρ_max_ = 1.76 e Å^−^^3^
711 parameters	Δρ_min_ = −0.67 e Å^−^^3^
0 restraints	

### Diisoselenocyanatotetrakis(2-methylpyridine N-oxide)cobalt(II) (2) Special details

**Table:**

Geometry. All esds (except the esd in the dihedral angle between two l.s. planes) are estimated using the full covariance matrix. The cell esds are taken into account individually in the estimation of esds in distances, angles and torsion angles; correlations between esds in cell parameters are only used when they are defined by crystal symmetry. An approximate (isotropic) treatment of cell esds is used for estimating esds involving l.s. planes.

### Diisoselenocyanatotetrakis(2-methylpyridine N-oxide)cobalt(II) (2) Fractional atomic coordinates and isotropic or equivalent isotropic displacement parameters (Å^2^)

**Table:**

	*x*	*y*	*z*	*U*_iso_*/*U*_eq_	
Co1	0.85687 (5)	0.30073 (2)	0.25349 (3)	0.03255 (11)	
N1	0.7045 (3)	0.30528 (7)	0.32998 (18)	0.0439 (6)	
C1	0.6211 (3)	0.30086 (8)	0.37078 (19)	0.0398 (6)	
Se1	0.48879 (4)	0.29344 (2)	0.43375 (2)	0.04996 (10)	
N2	0.8337 (3)	0.35121 (7)	0.21658 (19)	0.0467 (6)	
C2	0.7640 (3)	0.37377 (7)	0.18443 (19)	0.0375 (6)	
Se2	0.65659 (3)	0.40942 (2)	0.13745 (2)	0.04001 (9)	
O11	0.6964 (2)	0.29141 (6)	0.13849 (13)	0.0414 (5)	
N11	0.5621 (2)	0.27903 (6)	0.12825 (15)	0.0346 (5)	
C11	0.4567 (3)	0.28858 (8)	0.05670 (19)	0.0398 (6)	
C12	0.3196 (3)	0.27392 (8)	0.0426 (2)	0.0431 (7)	
H12	0.246440	0.279411	−0.008091	0.052*	
C13	0.2868 (3)	0.25143 (8)	0.1007 (2)	0.0465 (7)	
H13	0.191510	0.241859	0.091308	0.056*	
C14	0.3960 (3)	0.24317 (8)	0.1730 (2)	0.0447 (7)	
H14	0.375413	0.227885	0.213985	0.054*	
C15	0.5335 (3)	0.25683 (7)	0.1859 (2)	0.0390 (6)	
H15	0.608527	0.250736	0.235095	0.047*	
C16	0.4994 (4)	0.31461 (11)	0.0004 (2)	0.0534 (9)	
H16A	0.535110	0.334590	0.034815	0.080*	
H16B	0.413754	0.320524	−0.046059	0.080*	
H16C	0.577394	0.305811	−0.024481	0.080*	
O21	0.8669 (2)	0.25020 (6)	0.28634 (16)	0.0469 (5)	
N21	0.9700 (3)	0.23026 (6)	0.27081 (16)	0.0373 (5)	
C21	0.9424 (5)	0.20957 (10)	0.2003 (2)	0.0566 (9)	
C22	1.0556 (6)	0.18664 (9)	0.1935 (3)	0.0640 (12)	
H22	1.038070	0.171341	0.146527	0.077*	
C23	1.1853 (5)	0.18606 (10)	0.2511 (3)	0.0663 (11)	
H23	1.259130	0.170516	0.245622	0.080*	
C24	1.2092 (5)	0.20788 (11)	0.3168 (3)	0.0611 (10)	
H24	1.302134	0.208032	0.357349	0.073*	
C25	1.1028 (4)	0.22996 (9)	0.3268 (2)	0.0445 (7)	
H25	1.123087	0.245224	0.373844	0.053*	
C26	0.8030 (5)	0.21089 (12)	0.1372 (3)	0.0676 (11)	
H26A	0.724731	0.203455	0.163751	0.101*	
H26B	0.806154	0.196186	0.088647	0.101*	
H26C	0.783446	0.233942	0.116457	0.101*	
O31	1.0078 (2)	0.28863 (5)	0.18030 (12)	0.0339 (4)	
N31	1.1007 (2)	0.31146 (6)	0.16405 (14)	0.0305 (4)	
C31	1.0651 (3)	0.32881 (7)	0.08905 (17)	0.0332 (5)	
C32	1.1666 (3)	0.35134 (7)	0.07174 (19)	0.0370 (6)	
H32	1.142666	0.363892	0.019694	0.044*	
C33	1.3017 (3)	0.35587 (7)	0.12868 (19)	0.0373 (6)	
H33	1.370726	0.371318	0.116247	0.045*	
C34	1.3349 (3)	0.33734 (7)	0.20473 (19)	0.0360 (6)	
H34	1.427367	0.339968	0.244894	0.043*	
C35	1.2331 (3)	0.31525 (7)	0.22133 (18)	0.0337 (5)	
H35	1.255339	0.302541	0.273140	0.040*	
C36	0.9211 (3)	0.32107 (9)	0.02853 (19)	0.0432 (7)	
H36A	0.916201	0.297105	0.014824	0.065*	
H36B	0.911002	0.334061	−0.024614	0.065*	
H36C	0.841353	0.326956	0.055521	0.065*	
O41	1.0443 (3)	0.31159 (7)	0.35059 (15)	0.0514 (6)	
N41	1.0439 (3)	0.32764 (7)	0.42399 (16)	0.0446 (6)	
C41	1.1138 (4)	0.35725 (9)	0.4411 (2)	0.0446 (7)	
C42	1.1179 (4)	0.37286 (9)	0.5191 (2)	0.0526 (8)	
H42	1.169421	0.393427	0.532417	0.063*	
C43	1.0484 (5)	0.35906 (10)	0.5778 (2)	0.0624 (10)	
H43	1.050575	0.370045	0.630794	0.075*	
C44	0.9753 (5)	0.32870 (10)	0.5574 (2)	0.0605 (10)	
H44	0.925918	0.318749	0.596364	0.073*	
C45	0.9748 (4)	0.31314 (10)	0.4806 (2)	0.0512 (8)	
H45	0.926239	0.292243	0.466866	0.061*	
C46	1.1844 (4)	0.37162 (10)	0.3753 (2)	0.0529 (8)	
H46A	1.268379	0.357690	0.371152	0.079*	
H46B	1.218275	0.394423	0.392139	0.079*	
H46C	1.112834	0.372203	0.319137	0.079*	
Co2	0.05490 (5)	0.55048 (2)	0.26790 (3)	0.03372 (11)	
N3	−0.1122 (3)	0.55122 (7)	0.33403 (17)	0.0399 (5)	
C3	−0.1889 (3)	0.54722 (7)	0.37930 (19)	0.0366 (6)	
Se3	−0.30340 (4)	0.54154 (2)	0.45399 (2)	0.04069 (9)	
N4	0.0266 (3)	0.60176 (7)	0.23483 (17)	0.0417 (6)	
C4	−0.0233 (3)	0.62762 (8)	0.21203 (18)	0.0364 (6)	
Se4	−0.10390 (4)	0.66802 (2)	0.17864 (2)	0.04073 (9)	
O51	−0.0877 (2)	0.53873 (6)	0.14900 (14)	0.0443 (5)	
N51	−0.2280 (3)	0.53048 (6)	0.13995 (16)	0.0372 (5)	
C51	−0.3329 (3)	0.54714 (7)	0.08062 (19)	0.0374 (6)	
C52	−0.4772 (3)	0.53760 (8)	0.06833 (19)	0.0385 (6)	
H52	−0.550788	0.548674	0.025880	0.046*	
C53	−0.5174 (3)	0.51215 (8)	0.1167 (2)	0.0413 (6)	
H53	−0.617456	0.505829	0.108496	0.050*	
C54	−0.4072 (4)	0.49608 (8)	0.1776 (2)	0.0425 (7)	
H54	−0.431603	0.478620	0.211947	0.051*	
C55	−0.2633 (4)	0.50544 (8)	0.1879 (2)	0.0424 (7)	
H55	−0.188053	0.494262	0.229107	0.051*	
C56	−0.2814 (4)	0.57487 (9)	0.0324 (2)	0.0527 (8)	
H56A	−0.224618	0.591042	0.073505	0.079*	
H56B	−0.366620	0.586115	−0.004755	0.079*	
H56C	−0.219314	0.565636	−0.003085	0.079*	
O61	0.0747 (2)	0.49913 (5)	0.29973 (13)	0.0359 (4)	
N61	0.1925 (3)	0.48096 (6)	0.29677 (15)	0.0338 (5)	
C61	0.1780 (4)	0.45543 (9)	0.2397 (2)	0.0499 (8)	
C62	0.2985 (4)	0.43517 (9)	0.2418 (3)	0.0577 (9)	
H62	0.289413	0.417190	0.201929	0.069*	
C63	0.4301 (4)	0.44036 (8)	0.2998 (2)	0.0482 (8)	
H63	0.510759	0.425707	0.302108	0.058*	
C64	0.4434 (4)	0.46748 (9)	0.3553 (2)	0.0456 (7)	
H64	0.534361	0.472043	0.395294	0.055*	
C65	0.3234 (3)	0.48773 (8)	0.35187 (19)	0.0401 (6)	
H65	0.332637	0.506706	0.388678	0.048*	
C66	0.0321 (5)	0.45120 (16)	0.1783 (4)	0.098 (2)	
H66A	−0.041594	0.445915	0.210397	0.147*	
H66B	0.036981	0.432891	0.138290	0.147*	
H66C	0.004750	0.472002	0.145805	0.147*	
O71	0.2211 (2)	0.54389 (5)	0.20202 (13)	0.0365 (4)	
N71	0.3090 (3)	0.56936 (6)	0.19452 (15)	0.0340 (5)	
C71	0.2769 (3)	0.58840 (7)	0.12162 (18)	0.0362 (6)	
C72	0.3756 (3)	0.61346 (8)	0.1133 (2)	0.0403 (6)	
H72	0.355116	0.627086	0.063102	0.048*	
C73	0.5027 (3)	0.61885 (8)	0.1768 (2)	0.0409 (6)	
H73	0.569491	0.636000	0.170536	0.049*	
C74	0.5312 (3)	0.59888 (8)	0.2498 (2)	0.0402 (6)	
H74	0.618290	0.602127	0.294180	0.048*	
C75	0.4328 (3)	0.57434 (7)	0.25761 (18)	0.0364 (6)	
H75	0.451851	0.560697	0.307813	0.044*	
C76	0.1424 (3)	0.57984 (9)	0.0544 (2)	0.0442 (7)	
H76A	0.149950	0.556819	0.034623	0.066*	
H76B	0.131994	0.595263	0.005540	0.066*	
H76C	0.056233	0.581794	0.078453	0.066*	
O81	0.2256 (2)	0.56159 (5)	0.37630 (13)	0.0377 (4)	
N81	0.2060 (3)	0.56779 (6)	0.45506 (15)	0.0344 (5)	
C81	0.2607 (4)	0.59684 (8)	0.4944 (2)	0.0445 (7)	
C82	0.2398 (4)	0.60311 (9)	0.5759 (2)	0.0532 (8)	
H82	0.275400	0.623445	0.604265	0.064*	
C83	0.1686 (4)	0.58051 (9)	0.6165 (2)	0.0493 (8)	
H83	0.154145	0.585270	0.672016	0.059*	
C84	0.1182 (3)	0.55068 (8)	0.57515 (19)	0.0414 (7)	
H84	0.070081	0.534572	0.602321	0.050*	
C85	0.1390 (3)	0.54478 (7)	0.49424 (19)	0.0361 (6)	
H85	0.105963	0.524319	0.465635	0.043*	
C86	0.3388 (5)	0.61941 (10)	0.4461 (3)	0.0604 (10)	
H86A	0.425429	0.607893	0.436153	0.091*	
H86B	0.369744	0.639797	0.479800	0.091*	
H86C	0.272588	0.625365	0.390545	0.091*	

### Diisoselenocyanatotetrakis(2-methylpyridine N-oxide)cobalt(II) (2) Atomic displacement parameters (Å^2^)

**Table:**

	*U*^11^	*U*^22^	*U*^33^	*U*^12^	*U*^13^	*U*^23^
Co1	0.0293 (2)	0.0352 (2)	0.0326 (2)	−0.00107 (18)	0.00656 (18)	−0.00012 (18)
N1	0.0374 (14)	0.0524 (15)	0.0443 (14)	−0.0012 (11)	0.0144 (11)	−0.0022 (12)
C1	0.0345 (15)	0.0467 (16)	0.0352 (14)	0.0014 (12)	0.0029 (12)	−0.0006 (12)
Se1	0.03537 (18)	0.0772 (3)	0.03717 (17)	0.00101 (16)	0.00861 (13)	0.00799 (16)
N2	0.0508 (16)	0.0349 (13)	0.0565 (16)	0.0015 (12)	0.0173 (13)	0.0009 (12)
C2	0.0350 (15)	0.0401 (15)	0.0394 (14)	−0.0072 (12)	0.0130 (12)	−0.0054 (12)
Se2	0.03332 (16)	0.04557 (17)	0.03953 (16)	0.00037 (12)	0.00586 (12)	0.00369 (13)
O11	0.0252 (10)	0.0594 (13)	0.0384 (10)	−0.0092 (9)	0.0053 (8)	0.0014 (9)
N11	0.0258 (11)	0.0396 (12)	0.0383 (12)	−0.0030 (9)	0.0080 (9)	−0.0060 (10)
C11	0.0323 (15)	0.0492 (16)	0.0377 (14)	−0.0002 (12)	0.0081 (11)	−0.0080 (13)
C12	0.0297 (15)	0.0510 (17)	0.0459 (16)	0.0020 (12)	0.0039 (12)	−0.0121 (14)
C13	0.0300 (15)	0.0416 (16)	0.067 (2)	−0.0044 (12)	0.0099 (14)	−0.0127 (15)
C14	0.0352 (16)	0.0343 (14)	0.065 (2)	−0.0014 (12)	0.0143 (14)	−0.0021 (14)
C15	0.0295 (14)	0.0368 (14)	0.0508 (17)	0.0009 (11)	0.0098 (12)	−0.0024 (12)
C16	0.0357 (16)	0.084 (3)	0.0388 (16)	−0.0021 (16)	0.0054 (13)	0.0087 (16)
O21	0.0314 (11)	0.0444 (12)	0.0667 (15)	0.0046 (9)	0.0153 (10)	0.0183 (11)
N21	0.0359 (13)	0.0325 (11)	0.0430 (13)	−0.0004 (9)	0.0087 (10)	0.0075 (10)
C21	0.064 (2)	0.056 (2)	0.0451 (18)	−0.0266 (18)	0.0046 (16)	0.0075 (15)
C22	0.094 (3)	0.0383 (17)	0.076 (3)	−0.0175 (19)	0.053 (3)	−0.0129 (17)
C23	0.079 (3)	0.049 (2)	0.082 (3)	0.013 (2)	0.042 (2)	0.018 (2)
C24	0.058 (2)	0.075 (3)	0.055 (2)	0.0298 (19)	0.0229 (18)	0.0284 (19)
C25	0.0399 (17)	0.0553 (18)	0.0366 (15)	0.0067 (14)	0.0061 (12)	0.0069 (13)
C26	0.050 (2)	0.074 (3)	0.078 (3)	−0.0026 (19)	0.014 (2)	−0.016 (2)
O31	0.0311 (10)	0.0344 (9)	0.0371 (10)	−0.0043 (8)	0.0099 (8)	−0.0001 (8)
N31	0.0272 (11)	0.0329 (11)	0.0316 (11)	−0.0007 (9)	0.0078 (9)	−0.0018 (9)
C31	0.0292 (13)	0.0383 (14)	0.0315 (13)	0.0015 (11)	0.0066 (10)	−0.0002 (11)
C32	0.0371 (15)	0.0378 (14)	0.0376 (14)	0.0008 (11)	0.0120 (11)	0.0037 (11)
C33	0.0342 (14)	0.0367 (14)	0.0434 (15)	−0.0029 (11)	0.0143 (12)	−0.0024 (12)
C34	0.0285 (14)	0.0414 (14)	0.0372 (14)	−0.0016 (11)	0.0062 (11)	−0.0062 (11)
C35	0.0289 (13)	0.0385 (14)	0.0321 (13)	0.0028 (11)	0.0042 (10)	0.0004 (11)
C36	0.0333 (15)	0.0616 (19)	0.0323 (14)	−0.0040 (13)	0.0036 (11)	0.0033 (13)
O41	0.0393 (12)	0.0758 (16)	0.0382 (11)	−0.0101 (11)	0.0080 (9)	−0.0183 (11)
N41	0.0452 (15)	0.0552 (16)	0.0322 (12)	−0.0049 (12)	0.0074 (11)	−0.0044 (11)
C41	0.0459 (17)	0.0533 (18)	0.0338 (14)	−0.0029 (14)	0.0082 (12)	0.0004 (13)
C42	0.066 (2)	0.0513 (19)	0.0411 (17)	−0.0090 (17)	0.0142 (16)	−0.0054 (14)
C43	0.093 (3)	0.062 (2)	0.0349 (16)	−0.011 (2)	0.0193 (18)	−0.0065 (15)
C44	0.082 (3)	0.062 (2)	0.0416 (18)	−0.009 (2)	0.0224 (18)	0.0056 (16)
C45	0.056 (2)	0.058 (2)	0.0395 (16)	−0.0083 (16)	0.0112 (14)	0.0010 (15)
C46	0.052 (2)	0.068 (2)	0.0418 (17)	−0.0070 (17)	0.0171 (15)	−0.0013 (16)
Co2	0.0298 (2)	0.0371 (2)	0.0348 (2)	0.00232 (18)	0.00881 (18)	0.00530 (18)
N3	0.0324 (13)	0.0460 (14)	0.0431 (13)	0.0058 (10)	0.0126 (11)	0.0073 (11)
C3	0.0332 (14)	0.0352 (14)	0.0396 (14)	0.0032 (11)	0.0057 (12)	0.0050 (11)
Se3	0.03533 (17)	0.04521 (18)	0.04398 (17)	0.00154 (13)	0.01443 (13)	0.00795 (13)
N4	0.0429 (14)	0.0413 (13)	0.0428 (13)	0.0078 (11)	0.0143 (11)	0.0071 (11)
C4	0.0339 (14)	0.0448 (15)	0.0315 (13)	−0.0039 (12)	0.0101 (11)	0.0007 (11)
Se4	0.04327 (18)	0.03789 (16)	0.03780 (16)	−0.00041 (13)	0.00378 (13)	0.00388 (12)
O51	0.0297 (10)	0.0599 (13)	0.0411 (11)	−0.0058 (9)	0.0044 (8)	0.0130 (10)
N51	0.0299 (12)	0.0425 (13)	0.0391 (12)	−0.0036 (10)	0.0080 (9)	0.0048 (10)
C51	0.0354 (15)	0.0400 (14)	0.0363 (14)	−0.0005 (12)	0.0079 (11)	0.0040 (12)
C52	0.0336 (15)	0.0420 (15)	0.0391 (15)	0.0017 (12)	0.0075 (12)	−0.0036 (12)
C53	0.0349 (15)	0.0416 (15)	0.0512 (17)	−0.0037 (12)	0.0176 (13)	−0.0086 (13)
C54	0.0442 (17)	0.0371 (14)	0.0504 (17)	−0.0033 (12)	0.0196 (14)	0.0013 (13)
C55	0.0426 (17)	0.0418 (15)	0.0440 (16)	0.0023 (13)	0.0131 (13)	0.0090 (13)
C56	0.0430 (18)	0.060 (2)	0.0491 (18)	−0.0083 (15)	−0.0005 (14)	0.0199 (16)
O61	0.0310 (10)	0.0356 (10)	0.0424 (10)	0.0030 (8)	0.0114 (8)	0.0052 (8)
N61	0.0332 (12)	0.0338 (11)	0.0372 (12)	0.0012 (9)	0.0138 (9)	0.0042 (9)
C61	0.0402 (18)	0.0509 (18)	0.062 (2)	−0.0072 (14)	0.0186 (15)	−0.0162 (16)
C62	0.050 (2)	0.0446 (18)	0.085 (3)	−0.0064 (15)	0.0305 (19)	−0.0185 (18)
C63	0.0441 (18)	0.0400 (16)	0.069 (2)	0.0063 (13)	0.0302 (16)	0.0114 (15)
C64	0.0376 (16)	0.0554 (18)	0.0449 (16)	0.0079 (14)	0.0124 (13)	0.0098 (14)
C65	0.0368 (15)	0.0440 (15)	0.0380 (14)	0.0053 (12)	0.0067 (12)	0.0010 (12)
C66	0.043 (2)	0.128 (5)	0.116 (4)	−0.008 (3)	0.007 (2)	−0.080 (4)
O71	0.0330 (10)	0.0382 (10)	0.0408 (10)	−0.0011 (8)	0.0138 (8)	0.0027 (8)
N71	0.0307 (12)	0.0377 (12)	0.0358 (11)	0.0014 (9)	0.0123 (9)	0.0024 (9)
C71	0.0333 (14)	0.0413 (15)	0.0357 (14)	0.0042 (11)	0.0118 (11)	0.0021 (11)
C72	0.0393 (16)	0.0438 (15)	0.0394 (14)	0.0040 (12)	0.0127 (12)	0.0062 (12)
C73	0.0392 (16)	0.0392 (15)	0.0462 (16)	−0.0008 (12)	0.0142 (13)	0.0022 (13)
C74	0.0322 (15)	0.0482 (16)	0.0396 (15)	0.0016 (12)	0.0075 (12)	−0.0014 (13)
C75	0.0332 (14)	0.0406 (15)	0.0351 (13)	0.0044 (11)	0.0080 (11)	0.0040 (11)
C76	0.0359 (16)	0.0599 (19)	0.0373 (15)	−0.0003 (14)	0.0099 (12)	0.0044 (14)
O81	0.0350 (10)	0.0459 (11)	0.0331 (10)	−0.0007 (8)	0.0103 (8)	−0.0007 (8)
N81	0.0330 (12)	0.0372 (12)	0.0322 (11)	0.0029 (9)	0.0066 (9)	0.0020 (9)
C81	0.0486 (18)	0.0406 (15)	0.0414 (16)	−0.0075 (13)	0.0054 (13)	−0.0007 (13)
C82	0.066 (2)	0.0497 (18)	0.0386 (16)	−0.0079 (16)	0.0022 (15)	−0.0091 (14)
C83	0.054 (2)	0.061 (2)	0.0305 (14)	0.0028 (16)	0.0052 (13)	−0.0016 (14)
C84	0.0346 (15)	0.0523 (17)	0.0355 (14)	0.0044 (13)	0.0049 (12)	0.0092 (13)
C85	0.0314 (14)	0.0367 (14)	0.0382 (14)	0.0017 (11)	0.0046 (11)	0.0043 (11)
C86	0.073 (3)	0.0489 (19)	0.060 (2)	−0.0216 (18)	0.0177 (19)	−0.0026 (17)

### Diisoselenocyanatotetrakis(2-methylpyridine N-oxide)cobalt(II) (2) Geometric parameters (Å, º)

**Table:**

Co1—N1	2.105 (3)	Co2—N3	2.096 (3)
Co1—N2	2.096 (3)	Co2—N4	2.115 (3)
Co1—O11	2.104 (2)	Co2—O51	2.092 (2)
Co1—O21	2.081 (2)	Co2—O61	2.109 (2)
Co1—O31	2.103 (2)	Co2—O71	2.102 (2)
Co1—O41	2.086 (2)	Co2—O81	2.098 (2)
N1—C1	1.149 (4)	N3—C3	1.150 (4)
C1—Se1	1.805 (3)	C3—Se3	1.806 (3)
N2—C2	1.157 (4)	N4—C4	1.155 (4)
C2—Se2	1.798 (3)	C4—Se4	1.805 (3)
O11—N11	1.325 (3)	O51—N51	1.330 (3)
N11—C11	1.370 (4)	N51—C51	1.361 (4)
N11—C15	1.352 (4)	N51—C55	1.350 (4)
C11—C12	1.380 (4)	C51—C52	1.374 (4)
C11—C16	1.493 (5)	C51—C56	1.495 (4)
C12—H12	0.9500	C52—H52	0.9500
C12—C13	1.380 (5)	C52—C53	1.384 (4)
C13—H13	0.9500	C53—H53	0.9500
C13—C14	1.383 (5)	C53—C54	1.391 (5)
C14—H14	0.9500	C54—H54	0.9500
C14—C15	1.369 (4)	C54—C55	1.371 (5)
C15—H15	0.9500	C55—H55	0.9500
C16—H16A	0.9800	C56—H56A	0.9800
C16—H16B	0.9800	C56—H56B	0.9800
C16—H16C	0.9800	C56—H56C	0.9800
O21—N21	1.322 (3)	O61—N61	1.332 (3)
N21—C21	1.370 (5)	N61—C61	1.353 (4)
N21—C25	1.344 (4)	N61—C65	1.351 (4)
C21—C22	1.426 (6)	C61—C62	1.383 (5)
C21—C26	1.444 (6)	C61—C66	1.485 (6)
C22—H22	0.9500	C62—H62	0.9500
C22—C23	1.334 (7)	C62—C63	1.366 (6)
C23—H23	0.9500	C63—H63	0.9500
C23—C24	1.341 (6)	C63—C64	1.385 (5)
C24—H24	0.9500	C64—H64	0.9500
C24—C25	1.371 (5)	C64—C65	1.376 (4)
C25—H25	0.9500	C65—H65	0.9500
C26—H26A	0.9800	C66—H66A	0.9800
C26—H26B	0.9800	C66—H66B	0.9800
C26—H26C	0.9800	C66—H66C	0.9800
O31—N31	1.330 (3)	O71—N71	1.333 (3)
N31—C31	1.354 (4)	N71—C71	1.362 (4)
N31—C35	1.359 (3)	N71—C75	1.353 (4)
C31—C32	1.386 (4)	C71—C72	1.391 (4)
C31—C36	1.488 (4)	C71—C76	1.483 (4)
C32—H32	0.9500	C72—H72	0.9500
C32—C33	1.379 (4)	C72—C73	1.380 (4)
C33—H33	0.9500	C73—H73	0.9500
C33—C34	1.392 (4)	C73—C74	1.384 (4)
C34—H34	0.9500	C74—H74	0.9500
C34—C35	1.373 (4)	C74—C75	1.373 (4)
C35—H35	0.9500	C75—H75	0.9500
C36—H36A	0.9800	C76—H76A	0.9800
C36—H36B	0.9800	C76—H76B	0.9800
C36—H36C	0.9800	C76—H76C	0.9800
O41—N41	1.338 (3)	O81—N81	1.340 (3)
N41—C41	1.347 (4)	N81—C81	1.358 (4)
N41—C45	1.364 (4)	N81—C85	1.351 (4)
C41—C42	1.386 (4)	C81—C82	1.387 (5)
C41—C46	1.490 (5)	C81—C86	1.491 (5)
C42—H42	0.9500	C82—H82	0.9500
C42—C43	1.382 (5)	C82—C83	1.377 (5)
C43—H43	0.9500	C83—H83	0.9500
C43—C44	1.392 (6)	C83—C84	1.387 (5)
C44—H44	0.9500	C84—H84	0.9500
C44—C45	1.376 (5)	C84—C85	1.375 (4)
C45—H45	0.9500	C85—H85	0.9500
C46—H46A	0.9800	C86—H86A	0.9800
C46—H46B	0.9800	C86—H86B	0.9800
C46—H46C	0.9800	C86—H86C	0.9800
			
N2—Co1—N1	92.43 (11)	N3—Co2—N4	92.80 (10)
N2—Co1—O11	85.49 (11)	N3—Co2—O61	85.67 (9)
N2—Co1—O31	95.84 (10)	N3—Co2—O71	173.62 (9)
O11—Co1—N1	94.63 (10)	N3—Co2—O81	95.39 (9)
O21—Co1—N1	86.10 (10)	O51—Co2—N3	94.08 (10)
O21—Co1—N2	176.62 (11)	O51—Co2—N4	88.66 (10)
O21—Co1—O11	91.58 (10)	O51—Co2—O61	90.02 (9)
O21—Co1—O31	85.57 (8)	O51—Co2—O71	84.49 (9)
O21—Co1—O41	92.05 (10)	O51—Co2—O81	170.51 (9)
O31—Co1—N1	171.62 (10)	O61—Co2—N4	177.90 (9)
O31—Co1—O11	84.73 (8)	O71—Co2—N4	93.37 (9)
O41—Co1—N1	96.79 (10)	O71—Co2—O61	88.12 (8)
O41—Co1—N2	91.15 (12)	O81—Co2—N4	91.46 (10)
O41—Co1—O11	168.23 (9)	O81—Co2—O61	90.12 (8)
O41—Co1—O31	84.39 (8)	O81—Co2—O71	86.03 (8)
C1—N1—Co1	166.2 (3)	C3—N3—Co2	167.6 (2)
N1—C1—Se1	179.1 (3)	N3—C3—Se3	177.7 (3)
C2—N2—Co1	151.9 (3)	C4—N4—Co2	163.8 (3)
N2—C2—Se2	178.3 (3)	N4—C4—Se4	178.5 (3)
N11—O11—Co1	128.93 (17)	N51—O51—Co2	123.32 (18)
O11—N11—C11	118.0 (2)	O51—N51—C51	118.8 (2)
O11—N11—C15	120.1 (2)	O51—N51—C55	119.7 (2)
C15—N11—C11	121.9 (3)	C55—N51—C51	121.5 (3)
N11—C11—C12	118.1 (3)	N51—C51—C52	118.9 (3)
N11—C11—C16	116.8 (3)	N51—C51—C56	116.8 (3)
C12—C11—C16	125.0 (3)	C52—C51—C56	124.3 (3)
C11—C12—H12	119.4	C51—C52—H52	119.4
C13—C12—C11	121.2 (3)	C51—C52—C53	121.2 (3)
C13—C12—H12	119.4	C53—C52—H52	119.4
C12—C13—H13	120.8	C52—C53—H53	120.9
C12—C13—C14	118.4 (3)	C52—C53—C54	118.2 (3)
C14—C13—H13	120.8	C54—C53—H53	120.9
C13—C14—H14	119.7	C53—C54—H54	120.0
C15—C14—C13	120.6 (3)	C55—C54—C53	120.0 (3)
C15—C14—H14	119.7	C55—C54—H54	120.0
N11—C15—C14	119.7 (3)	N51—C55—C54	120.3 (3)
N11—C15—H15	120.1	N51—C55—H55	119.9
C14—C15—H15	120.1	C54—C55—H55	119.9
C11—C16—H16A	109.5	C51—C56—H56A	109.5
C11—C16—H16B	109.5	C51—C56—H56B	109.5
C11—C16—H16C	109.5	C51—C56—H56C	109.5
H16A—C16—H16B	109.5	H56A—C56—H56B	109.5
H16A—C16—H16C	109.5	H56A—C56—H56C	109.5
H16B—C16—H16C	109.5	H56B—C56—H56C	109.5
N21—O21—Co1	121.76 (17)	N61—O61—Co2	123.03 (16)
O21—N21—C21	121.3 (3)	O61—N61—C61	119.2 (3)
O21—N21—C25	118.7 (3)	O61—N61—C65	119.8 (2)
C25—N21—C21	120.0 (3)	C65—N61—C61	121.0 (3)
N21—C21—C22	117.2 (3)	N61—C61—C62	118.5 (3)
N21—C21—C26	120.6 (4)	N61—C61—C66	117.1 (3)
C22—C21—C26	122.3 (4)	C62—C61—C66	124.4 (3)
C21—C22—H22	119.1	C61—C62—H62	119.2
C23—C22—C21	121.9 (4)	C63—C62—C61	121.7 (3)
C23—C22—H22	119.1	C63—C62—H62	119.2
C22—C23—H23	120.7	C62—C63—H63	120.7
C22—C23—C24	118.6 (4)	C62—C63—C64	118.5 (3)
C24—C23—H23	120.7	C64—C63—H63	120.7
C23—C24—H24	119.2	C63—C64—H64	120.4
C23—C24—C25	121.5 (4)	C65—C64—C63	119.3 (3)
C25—C24—H24	119.2	C65—C64—H64	120.4
N21—C25—C24	120.7 (3)	N61—C65—C64	120.8 (3)
N21—C25—H25	119.6	N61—C65—H65	119.6
C24—C25—H25	119.6	C64—C65—H65	119.6
C21—C26—H26A	109.5	C61—C66—H66A	109.5
C21—C26—H26B	109.5	C61—C66—H66B	109.5
C21—C26—H26C	109.5	C61—C66—H66C	109.5
H26A—C26—H26B	109.5	H66A—C66—H66B	109.5
H26A—C26—H26C	109.5	H66A—C66—H66C	109.5
H26B—C26—H26C	109.5	H66B—C66—H66C	109.5
N31—O31—Co1	120.96 (15)	N71—O71—Co2	120.08 (16)
O31—N31—C31	119.5 (2)	O71—N71—C71	119.4 (2)
O31—N31—C35	118.6 (2)	O71—N71—C75	118.8 (2)
C31—N31—C35	121.7 (2)	C75—N71—C71	121.7 (3)
N31—C31—C32	118.5 (3)	N71—C71—C72	118.0 (3)
N31—C31—C36	117.2 (3)	N71—C71—C76	117.5 (3)
C32—C31—C36	124.3 (3)	C72—C71—C76	124.4 (3)
C31—C32—H32	119.4	C71—C72—H72	119.4
C33—C32—C31	121.2 (3)	C73—C72—C71	121.2 (3)
C33—C32—H32	119.4	C73—C72—H72	119.4
C32—C33—H33	120.7	C72—C73—H73	120.5
C32—C33—C34	118.7 (3)	C72—C73—C74	118.9 (3)
C34—C33—H33	120.7	C74—C73—H73	120.5
C33—C34—H34	120.2	C73—C74—H74	120.3
C35—C34—C33	119.6 (3)	C75—C74—C73	119.5 (3)
C35—C34—H34	120.2	C75—C74—H74	120.3
N31—C35—C34	120.4 (3)	N71—C75—C74	120.7 (3)
N31—C35—H35	119.8	N71—C75—H75	119.6
C34—C35—H35	119.8	C74—C75—H75	119.6
C31—C36—H36A	109.5	C71—C76—H76A	109.5
C31—C36—H36B	109.5	C71—C76—H76B	109.5
C31—C36—H36C	109.5	C71—C76—H76C	109.5
H36A—C36—H36B	109.5	H76A—C76—H76B	109.5
H36A—C36—H36C	109.5	H76A—C76—H76C	109.5
H36B—C36—H36C	109.5	H76B—C76—H76C	109.5
N41—O41—Co1	124.68 (19)	N81—O81—Co2	124.24 (16)
O41—N41—C41	119.5 (3)	O81—N81—C81	118.0 (2)
O41—N41—C45	118.9 (3)	O81—N81—C85	119.8 (2)
C41—N41—C45	121.6 (3)	C85—N81—C81	122.1 (3)
N41—C41—C42	118.8 (3)	N81—C81—C82	117.7 (3)
N41—C41—C46	118.2 (3)	N81—C81—C86	117.0 (3)
C42—C41—C46	123.0 (3)	C82—C81—C86	125.4 (3)
C41—C42—H42	119.4	C81—C82—H82	119.3
C43—C42—C41	121.2 (3)	C83—C82—C81	121.5 (3)
C43—C42—H42	119.4	C83—C82—H82	119.3
C42—C43—H43	120.8	C82—C83—H83	120.5
C42—C43—C44	118.4 (3)	C82—C83—C84	119.1 (3)
C44—C43—H43	120.8	C84—C83—H83	120.5
C43—C44—H44	120.1	C83—C84—H84	120.5
C45—C44—C43	119.7 (3)	C85—C84—C83	119.0 (3)
C45—C44—H44	120.1	C85—C84—H84	120.5
N41—C45—C44	120.2 (3)	N81—C85—C84	120.6 (3)
N41—C45—H45	119.9	N81—C85—H85	119.7
C44—C45—H45	119.9	C84—C85—H85	119.7
C41—C46—H46A	109.5	C81—C86—H86A	109.5
C41—C46—H46B	109.5	C81—C86—H86B	109.5
C41—C46—H46C	109.5	C81—C86—H86C	109.5
H46A—C46—H46B	109.5	H86A—C86—H86B	109.5
H46A—C46—H46C	109.5	H86A—C86—H86C	109.5
H46B—C46—H46C	109.5	H86B—C86—H86C	109.5
			
Co1—O11—N11—C11	151.0 (2)	Co2—O51—N51—C51	128.0 (2)
Co1—O11—N11—C15	−31.1 (4)	Co2—O51—N51—C55	−53.5 (4)
Co1—O21—N21—C21	−99.2 (3)	Co2—O61—N61—C61	−115.5 (3)
Co1—O21—N21—C25	82.3 (3)	Co2—O61—N61—C65	66.0 (3)
Co1—O31—N31—C31	−95.3 (2)	Co2—O71—N71—C71	−95.9 (3)
Co1—O31—N31—C35	88.6 (3)	Co2—O71—N71—C75	88.0 (3)
Co1—O41—N41—C41	−117.7 (3)	Co2—O81—N81—C81	−125.5 (2)
Co1—O41—N41—C45	63.6 (4)	Co2—O81—N81—C85	57.2 (3)
O11—N11—C11—C12	175.4 (3)	O51—N51—C51—C52	177.0 (3)
O11—N11—C11—C16	−5.4 (4)	O51—N51—C51—C56	−2.7 (4)
O11—N11—C15—C14	−177.4 (3)	O51—N51—C55—C54	−178.2 (3)
N11—C11—C12—C13	3.0 (5)	N51—C51—C52—C53	1.8 (5)
C11—N11—C15—C14	0.3 (4)	C51—N51—C55—C54	0.2 (5)
C11—C12—C13—C14	−1.6 (5)	C51—C52—C53—C54	−0.8 (5)
C12—C13—C14—C15	−0.5 (5)	C52—C53—C54—C55	−0.4 (5)
C13—C14—C15—N11	1.1 (5)	C53—C54—C55—N51	0.7 (5)
C15—N11—C11—C12	−2.4 (4)	C55—N51—C51—C52	−1.5 (5)
C15—N11—C11—C16	176.8 (3)	C55—N51—C51—C56	178.8 (3)
C16—C11—C12—C13	−176.1 (3)	C56—C51—C52—C53	−178.5 (3)
O21—N21—C21—C22	−174.0 (3)	O61—N61—C61—C62	−175.1 (3)
O21—N21—C21—C26	4.8 (5)	O61—N61—C61—C66	5.1 (5)
O21—N21—C25—C24	175.0 (3)	O61—N61—C65—C64	174.1 (3)
N21—C21—C22—C23	−2.6 (5)	N61—C61—C62—C63	0.1 (6)
C21—N21—C25—C24	−3.5 (5)	C61—N61—C65—C64	−4.3 (5)
C21—C22—C23—C24	−0.4 (6)	C61—C62—C63—C64	−2.5 (6)
C22—C23—C24—C25	1.5 (6)	C62—C63—C64—C65	1.7 (5)
C23—C24—C25—N21	0.4 (6)	C63—C64—C65—N61	1.7 (5)
C25—N21—C21—C22	4.5 (4)	C65—N61—C61—C62	3.4 (5)
C25—N21—C21—C26	−176.7 (3)	C65—N61—C61—C66	−176.5 (4)
C26—C21—C22—C23	178.6 (4)	C66—C61—C62—C63	179.9 (5)
O31—N31—C31—C32	−177.0 (2)	O71—N71—C71—C72	−176.0 (2)
O31—N31—C31—C36	0.1 (4)	O71—N71—C71—C76	1.0 (4)
O31—N31—C35—C34	176.7 (2)	O71—N71—C75—C74	175.7 (3)
N31—C31—C32—C33	0.8 (4)	N71—C71—C72—C73	0.2 (4)
C31—N31—C35—C34	0.7 (4)	C71—N71—C75—C74	−0.3 (4)
C31—C32—C33—C34	−0.2 (4)	C71—C72—C73—C74	−0.1 (5)
C32—C33—C34—C35	−0.2 (4)	C72—C73—C74—C75	−0.2 (5)
C33—C34—C35—N31	−0.1 (4)	C73—C74—C75—N71	0.4 (5)
C35—N31—C31—C32	−1.1 (4)	C75—N71—C71—C72	0.0 (4)
C35—N31—C31—C36	176.1 (3)	C75—N71—C71—C76	176.9 (3)
C36—C31—C32—C33	−176.2 (3)	C76—C71—C72—C73	−176.5 (3)
O41—N41—C41—C42	−177.2 (3)	O81—N81—C81—C82	179.9 (3)
O41—N41—C41—C46	2.4 (5)	O81—N81—C81—C86	−0.2 (4)
O41—N41—C45—C44	178.6 (3)	O81—N81—C85—C84	179.9 (3)
N41—C41—C42—C43	−1.8 (6)	N81—C81—C82—C83	1.2 (5)
C41—N41—C45—C44	0.0 (6)	C81—N81—C85—C84	2.7 (4)
C41—C42—C43—C44	0.8 (7)	C81—C82—C83—C84	0.7 (6)
C42—C43—C44—C45	0.5 (7)	C82—C83—C84—C85	−0.9 (5)
C43—C44—C45—N41	−1.0 (6)	C83—C84—C85—N81	−0.7 (4)
C45—N41—C41—C42	1.4 (5)	C85—N81—C81—C82	−2.9 (5)
C45—N41—C41—C46	−179.0 (3)	C85—N81—C81—C86	177.0 (3)
C46—C41—C42—C43	178.6 (4)	C86—C81—C82—C83	−178.7 (4)

### Diisoselenocyanatotetrakis(2-methylpyridine N-oxide)cobalt(II) (2) Hydrogen-bond geometry (Å, º)

**Table:**

*D*—H···*A*	*D*—H	H···*A*	*D*···*A*	*D*—H···*A*
C15—H15···O21	0.95	2.36	3.158 (4)	141
C26—H26*C*···O11	0.98	2.49	3.367 (5)	149
C35—H35···Se1^i^	0.95	2.96	3.748 (3)	141
C36—H36*C*···O11	0.98	2.55	3.281 (4)	131
C42—H42···Se3^ii^	0.95	2.87	3.811 (4)	171
C52—H52···Se2^iii^	0.95	3.05	3.926 (3)	153
C55—H55···O61	0.95	2.45	3.245 (4)	141
C56—H56*B*···Se2^iii^	0.98	3.02	3.933 (3)	156
C65—H65···O81	0.95	2.40	3.140 (4)	135
C66—H66*A*···Se2^iv^	0.98	3.14	3.807 (5)	126
C75—H75···Se3^i^	0.95	2.95	3.726 (3)	140
C75—H75···O81	0.95	2.62	3.075 (3)	110
C76—H76*C*···O51	0.98	2.61	3.354 (4)	133
C86—H86*B*···Se1^ii^	0.98	3.14	4.109 (4)	169

Symmetry codes: (i) *x*+1, *y*, *z*; (ii) −*x*+1, −*y*+1, −*z*+1; (iii) −*x*, −*y*+1, −*z*; (iv) *x*−1, *y*, *z*.
